# Lessons, connections, hypotheses and predictions from protein film electrochemistry

**DOI:** 10.1007/s00775-026-02136-1

**Published:** 2026-03-11

**Authors:** Fraser A. Armstrong

**Affiliations:** Department of Chemistry and St John’s College, University of Oxford, Oxford, OX1 3JP UK

**Keywords:** Protein film electrochemistry electron transfer electrocatalysis metalloenzymes

## Abstract

This extended essay describes the development of protein film electrochemistry (PFE) as a powerful suite of techniques for investigating how electron-transfer processes occurring in biological molecules are coupled to chemical reactions. Based on the author’s personal experiences, the article explores the wider picture through connections to related work and other scientific areas, as well as hypotheses and predictions. Extending far beyond its electrochemical origins and early expectations, PFE has reached a wide audience – across disciplines as diverse as electrochemistry, renewable energy, biological coordination chemistry, molecular catalysis, biocatalysis, molecular and cell biology, biotechnology and pharmacology. Much is owed to many researchers, from undergraduates to senior figures, with whom the author has worked alongside and been inspired by for more than 40 years.

**Graphical abstract:**

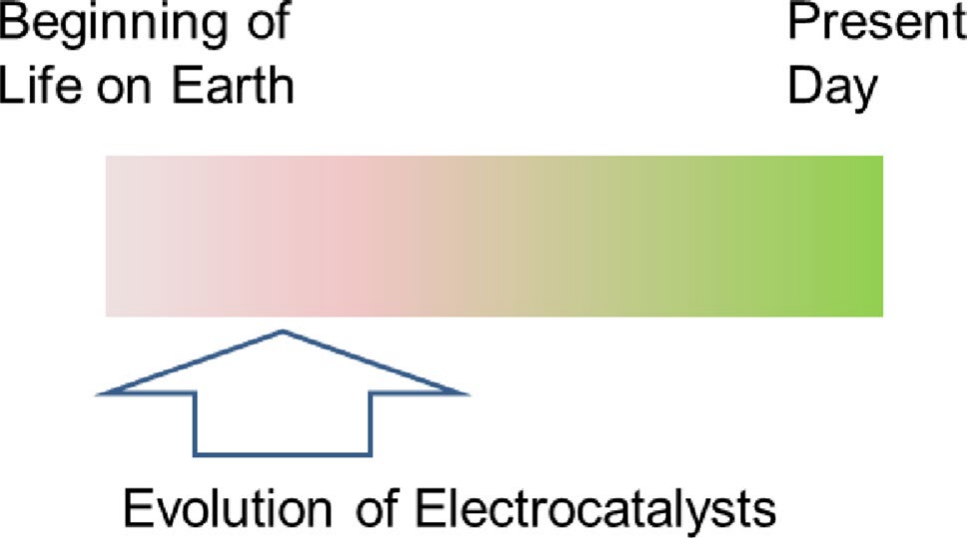

## Background

As a postdoc with Helmut Beinert at the Enzyme Institute in Madison, Wisconsin, I witnessed the meticulous detective work required to solve a problem that had not been encountered before. Although I was not directly involved in the group’s pioneering work on aconitase, an enzyme of the Tricarboxylic Acid (TCA) cycle, I realised the difficulty of pinning down the identity of an active site that keeps changing its character. Through old-fashioned and meticulous elemental analysis along with EPR spectroscopy, Helmut and his co-workers eventually established that the active site of aconitase is a [4Fe-4S] cluster that reverts to an inactive [3Fe-4S] cluster when oxidized, but re-assembles when Fe^2+^ and reducing conditions are restored. Later, I was very fortunate to join the group of Allen Hill in Oxford: Allen was looking for ways to exploit the direct electrochemistry of proteins, following the discovery of the reversible cyclic voltammetry of cytochrome c contained in solution. Like EPR, a voltammogram conveys a signal, and although a current/voltage signal is less useful than g-values as an identifying fingerprint, it is highly informative for real-time transformations. A casual conversation with Andrew Thomson at a conference in St Andrews in 1986 would lead me and others to develop protein film electrochemistry (PFE) as a powerful tool for studying active sites. Andrew’s group was grappling with Fe-S clusters in small proteins that appeared chaotic and unpredictable from one experiment to another – suggesting that aconitase-like vascillations of cluster structure were widespread.

**Fig. 1:**
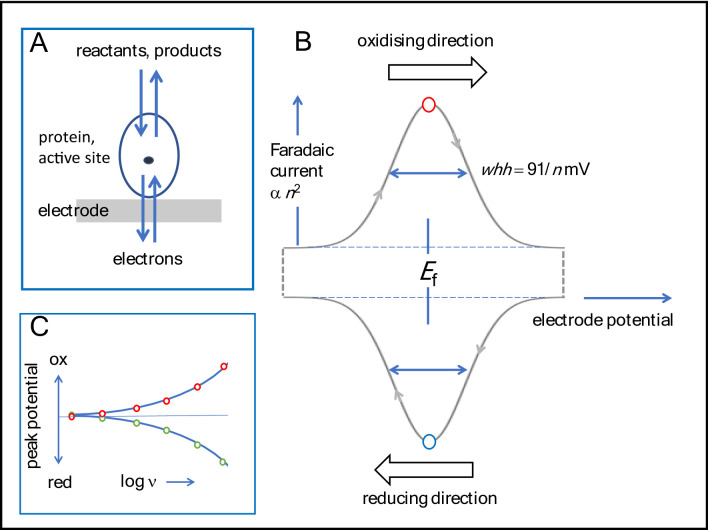
Basic aspects of the electron-exchange process in Protein Film Electrochemistry. **A** Cartoon showing protein attached to electrode surface. **B** The signal arising from non-catalytic electron exchange, indicating direction of cycling (arrowed). Terms: *n* = number of electrons transferred (where relevant, in a cooperatively-coupled process); *whh* = width at half height; *E*_f_ = formal potential. **C** Format of a ‘trumpet plot’ used to analyse electron-transfer kinetics and coupling, ν = scan rate

**Redox-active centres produce characteristic interactive electron-exchange signals.** During the following years, the chemistry of such vacillatory centres was uncovered, a crucial point being the early discovery that small proteins could be made to adsorb tightly yet innocuously on an electrode surface (Fig. 1A), and with fast *interfacial* electron tunnelling, their active sites were identified by compact, gaussian peak-type voltametric signals that revealed their reactivities when subjected to different stimuli (Fig. 1B).

Lacking a broad diffusion ‘tail’ (as there is no interference from slow mass-transport of protein molecules from solution) the voltammetry of proteins became much more useful; furthermore, with just minuscule sample requirements (often orders of magnitude lower than used for EPR spectroscopy) and the abilities to remove and replace reactants in solution, PFE would allow both trailblazing and detailed kinetic studies, each with highly interactive real-time experiments. While she was a graduate student with me at the University of California, Irvine (UCI) between 1989 and 1993, Julea Butt would uncover new aspects of the reactions of Fe-S clusters in proteins.

**Fig. 2:**
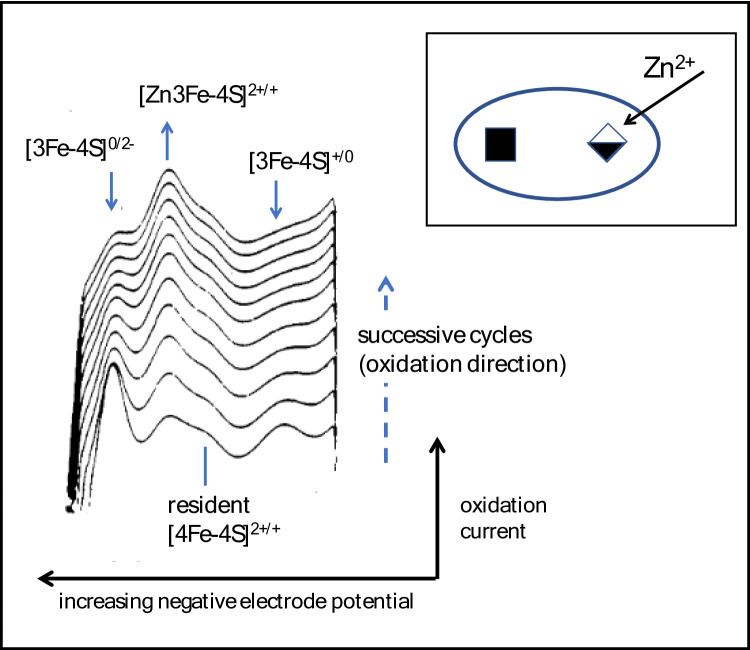
Insertion of a Zn^2+^ ion into a [3Fe-4S] cluster [1]. The subject is the 7Fe ferredoxin III from *Desulfovibrio africanus*: this protein contains a stable (resident) [4Fe-4S] cluster and a reactive [3Fe-4S] cluster. Only the oxidation direction is shown and scans are offset in the upward direction. Each cycle takes 3.6 s (scan rate = 470 mVs^− 1^), so the first (lower trace) corresponds to a reaction time of 2–3 s after injecting Zn^2+^ into the cell solution

An early success was ability to visualise, in real time, how a [3Fe-4S] cluster in certain proteins acts as a redox-switchable ligand – if exposed, the tripodal (µ_2_-S)_3_ face binds Fe^2+^ to form a [4Fe-4S] cubane or analogous products with many other metal ions [1–3]. As Dick Holm and co-workers noted, a [3Fe-4S] analog is extremely difficult to synthesize because the vacant subsite must be protected from other metal ions [4, 5]. Figure 2 illustrates how PFE was used to monitor the insertion of Zn^2+^ into a reactive [3Fe-4S] cluster over the course of several seconds after initiating the reaction by scanning from the positive potential limit [1].

One did not need to be an electrochemist to recognise, immediately, that the different Fe-S clusters in the protein are distinguished through their clear and specific electron-exchange signals. Unlike cytochromes, Fe-S centres are poorly differentiated by convenient UV-visible spectra, but EPR and MCD spectroscopy would subsequently complement the PFE trailblazing to characterise the products(s) formed in solution. Interconversions between different Fe-S clusters and related reactions are explained in more detail later.

Barbara Burgess, a close friend and colleague at UCI quickly recognised the value of PFE, and her support raised my confidence in extending it to explore wider fundamental properties of metalloprotein active sites, in particular the various ways that long-range electron transfer (ET) is coupled to proton transfer, ligand exchange and catalysis. With the signatures of active sites now displayed in both potential and time domains, PFE would offer new opportunities to control, drive and monitor these reactions in a simultaneous manner. Unlike conventional chemical methods used to study redox reactions of proteins, electrochemical methods use a continuous range of potentials and, with many electrodes, the useful potential window extends well outside the thermodynamic limits of water breakdown. The scene had already been set by Allen Bard and Marion Stankovich who in 1977 had studied insulin adsorbed at a mercury electrode, assigning signals due to the breaking and formation of S-S bonds, but noting the likelihood that direct Hg-S bonds play a non-innocent role [6]. Other researchers would focus on the adsorption and interfacial ET processes, i.e. accounting for how an electron-transferring protein becomes bound to an electrode surface and achieves efficient long-range electron tunnelling [7]. A logical starting point is that such proteins are naturally well equipped to do this, and at least one redox centre having a low reorganisation energy is located within 12Å of the protein surface [8–11]. The main consideration is for the protein to bind to the electrode surface and subsequently, through dynamic adjustments within that state, encounter a productive orientation. My group at UCI and later, Oxford, skipped over this issue – given that interfacial ET is fast and reversible, PFE should now provide fresh and alternative insight into the reactions of proteins and their active sites, with particular emphasis on metal cofactors. We envisaged that PFE would complement structural and spectroscopic information by introducing a dynamic electrochemical landscape that displays reactivity and rates as functions of potential (free energy) and time.

For a redox protein attached to an electrode and undergoing a cycle of oxidation and reduction during which *n* electrons are transferred, the electron-exchange signal is based on the waveshape (Fig. 1B) for a cyclic voltammogram clearly described by Etienne Laviron, consisting of a pair of oxidation and reduction peaks [12]. The main points that concern us are: (a) the basic thermodynamic rules pertaining to equilibrium (the Nernst equation) which predict that oxidation and reduction peaks each appear at the formal potential *E*_f_, the peak width at half height (*whh*) varies with 1/*n* (91/n mV at 20 °C) and the peak current varies as *n*^2^; (b) the charge passed during complete passage of each peak (equivalent to taking the integral) from which the number of electroactive sites and coverage are calculated; (c) the degree of dispersion (inhomogeneity among adsorbed protein molecules) which will tend to broaden a peak; (d) in the case where two electrons are transferred – the degree to which the one-electron component steps are coupled, since with strong cooperative coupling the first electron transfer assists the second [13, 14]. Cooperative two-electron transfers give much more prominent signals, greatly aiding the detection of active sites in larger proteins where coverage is low. The limiting case, producing a very prominent signal, is a fully cooperative two-electron transfer (*n* = 2, leading to *whh* ~ 45 mV and peak current 4-fold higher than *n* = 1) which occurs when *E*_2_ >> *E*_1_, in which case the two potentials are said to be highly *inverted* (*crossed*): this situation applies for nicotinamide NAD(P)(H), and flavin cofactors in many proteins. Cooperativity can involve two separate redox centres if they interact in a positive manner: for example, for two one-electron transfers having similar microscopic potentials (*E*_2_ ~ *E*_1_), the resulting cooperativity offsets the statistically-required separation of the two potentials to give a signal integrating to the passage of both electrons, with *whh* < 91 mV and peak current up to four times higher.

Even with cooperative enhancement, the magnitude of electron-exchange signals is usually very small compared to the electrode capacitance, and background subtraction is required to analyse them properly – a excellent tool being SOAS, software developed by Christophe Léger and coworkers) [15]. While the mainstay of PFE has been cyclic or linear-sweep voltammetry, supported by chronoamperometry (time-dependent observations at constant potential), we and others would expand the repertoire to include digitally-based methods such as square-wave voltammetry and Fourier transform voltammetry which offer greater sensitivity [16–21]. The suite of techniques available for PFE would thus reveal the signatures and behaviour of redox-active sites and complete enzymes in the potential, time and frequency domains. 

A key practical component was the electrode itself – a favourite material being a pyrolytic graphite disc cut so that the surface is perpendicular to the hydrophobic basal plane [22]. The resulting surface is rough and dotted with polar C-O functionalities. We referred to the electrode as a pyrolytic graphite edge (PGE) electrode, noting that the cut edge cannot be a true (atomically flat) plane, as misleadingly described by others. We found it convenient to apply protein molecules as a solution to the electrode surface, then rinse after allowing time for adsorption. It was essential that the electrode is innocent – i.e. it does not interfere with the protein’s function. Whereas metal electrodes such as mercury and gold have reactive surface atoms that form stable bonds to soft donors in protein molecules and potentially promote unfolding, an electrode in which the surface atoms have satisfied valencies, such as PGE, does not do so. In those early years I was inspired by papers by Jim Barber describing how Mg^2+^ ions promote the stacking of negatively charged thylakoid membranes [23]. Acting on these ideas, coulombic interactions and the inclusion of mobile counter ions (Mg^2+^, Cr(NH_3_)_6_^3+^, organic polycations) were established to be important for achieving the adsorption of many proteins at PGE. Other electrodes have included ones modified with carbon nanotubes or surfactant films, several metallic oxides, and Au (or Ag) modified with a monolayer of functionalised conducting organic molecules (applying ideas from Christopher Chidsey and co-workers) [24–26]. Enzyme molecules, particularly ones modified by site-directed mutagenesis to present a specific attachment point, have been covalently linked to electrode surfaces [27]. Taking PFE in a more biologically realistic direction, Lars Jeuken and his colleagues have developed electrodes that support membrane-like bilayers [28], and as described later, mesoporous metallic oxides underpin a further direction for bioinspired catalysis.

It soon became clear that interfacial ET with redox proteins could be fast and efficient. Referring to the pedagogical work by Laviron, as the voltametric scan rate increases, the oxidation and reduction peak potentials separate in a way that depends on the elementary rate constant *k*_0_ [12]. The results are displayed conveniently in ‘trumpet plots’ (Fig. 1C). In some cases, such as the small blue Cu protein Azurin, scan rates exceeding 1000 V s^− 1^ could be used. Azurin was a superb subject for detailed investigations of the interfacial electron-exchange process, in which we were interested in the rate constants *k*_0_ (the *exchange* rate constant modelled using the standard Butler-Volmer equation), *k*_max_ (the *limiting* value from Marcus theory) and the role of gating [24, 29, 30]. Lars Jeuken, then a postdoc, and graduate student James McEvoy extended the picture further using large-amplitude square-wave voltammetry [21].

## Time-dependent coupling of electron transfer and chemical reactions

One-electron transfers form the basis for many more elaborate and useful processes. Not only can one-electron transfers be coupled together, but electron transfers can be coupled with proton transfers or other changes in chemical structure. The coupled processes often occur on a slower time scale than elementary electron transfer. Documenting redox biochemistry has long relied on potentiometrically-determined reduction potentials (aka ‘mid-point potentials’) in which all states have equilibrated, but the data have much less meaning for dynamic systems – those in action and undergoing coupled reactions such as catalysis [31]. Alan Bond has been a pioneer in considering complex redox processes in terms of *square schemes*, which account for aspects of non-ideal behaviour in cyclic voltammetry by adding chemical steps, including reorganisation on a slower time-scale and thus beyond that embodied within Marcus theory [32]. If an elementary electron-transfer step **E** depends upon a preceding non-electrochemical (chemical) step **C**, the electrochemical consequence is that the observed rate does not depend on the electrode potential in the manner expected from basic theory: it is the simplest example of *gated* electron transfer.

**Fig. 3:**
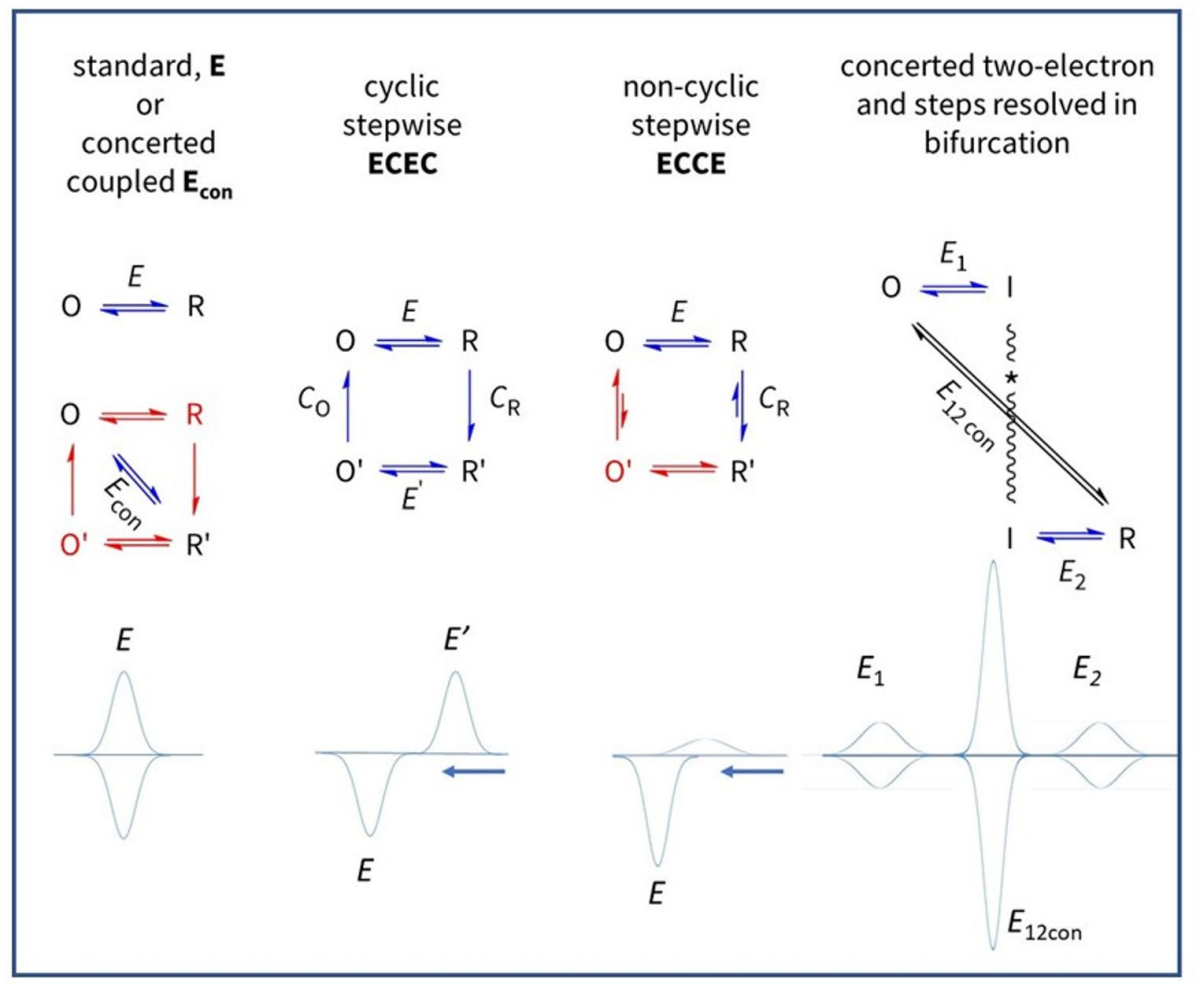
Square-scheme coupling patterns and corresponding PFE cyclic voltammograms. The processes are defined by the order of electron transfer (**E**) and chemical (**C**) steps driven as the electrode potential is cycled to interchange reduced R and oxidised O forms. In the standard case, a simple voltammogram is observed for a one-electron transfer (**E** alone) in which all atomic reorganisation is embodied in Marcus theory. Favoured pathways are shown in blue. A simple voltammogram is also obtained if electron transfer is coupled to a chemical step that is relatively rapid on the experimental timescale (E_diag_ combines the free energy changes of both steps). In a cyclic stepwise system **ECEC**, chemical steps follow each electron transfer. In non-cyclic stepwise **ECCE** a cyclic process is disfavoured, thus when the scan direction is reversed, the reverse chemical step gates electron transfer. A two-electron transfer is highly cooperative if **E**_1_ and **E**_2_ steps have highly inverted (crossed) potentials, i.e. *E*_2_ > > *E*_1_, so that intermediate I is unstable (in practical terms, the *n* = 2 limit is reached at Δ*E* ~ 180 mV) and may even proceed via a concerted mechanism. The two-electron voltammogram is flanked by its unobserved one-electron components. Kinetic separation of the two one-electron transfers affords the possibility of *bifurcation*, allowing an unfavourable one-electron reaction to occur by making the other much easier

With PFE, entire processes can be modelled or deconvoluted by analysing cyclic voltammograms measured over a range of conditions, particularly the scan rate. Unlike solution-based voltammetry, the result may differ greatly depending upon whether the cycle starts at the positive or negative potential limit. Some generic scenarios in which **E** and **C** steps may be stepwise or concerted are shown in Fig. 3. In each case, O and R states might also be connected by a non-electrochemical pathway – one that (for example) regenerates O when a reducing potential is applied to form R – thereby rendering the active site an electrocatalyst and resulting in a large continuous current (note that reactions catalysed by almost all redox enzymes require the transfer of two or more electrons). If **E** and **C** occur in a single ‘diagonal’ process (**C** being fast relative to the experimental timescale) a simple voltammogram is observed as for the standard case, best-known examples being where **C** refers to a fast proton transfer [33–36]. Concerted proton-coupled ET (concerted PCET) is highly relevant in enzyme catalysis and, as mentioned later, PFE can detect even subtle changes traceable to a minor modification (such as introduced by site-directed mutagenesis) that causes a concerted process to become stepwise.

**Fig. 4:**
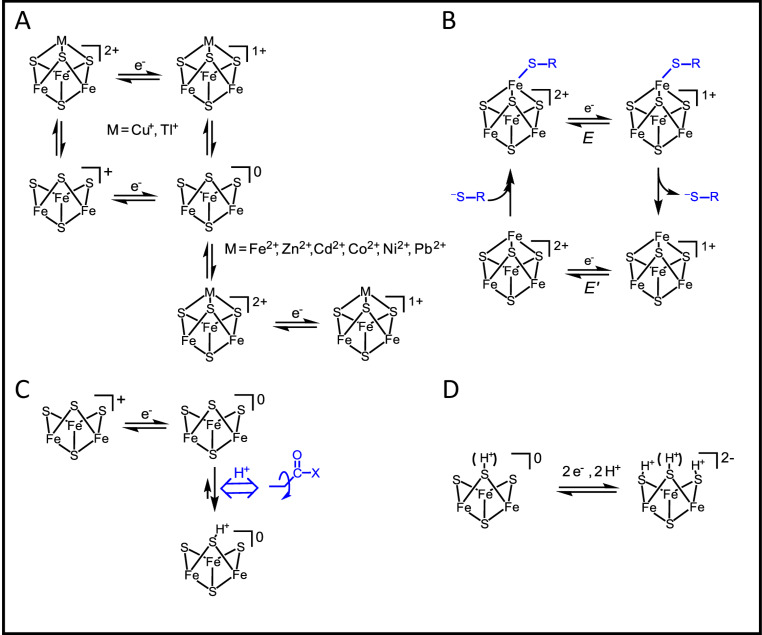
Reaction schemes for Fe-S clusters revealed and analysed by PFE (see text). **A** Reactions of an exposed [3Fe-4S] cluster with external metal ions. **B** Redox-level dependent binding of an external thiolate ligand (R-S^−^) to a [4Fe-4S] cluster. **C** Proton transfer at a one-electron reduced [3Fe-4S] cluster. **D** Generation of an all-Fe(II) [3Fe-4S] cluster supported by multiple protonation

Figure 4 shows some experimentally observed reactions of Fe-S clusters, each of which can be identified with scenarios shown in Fig. 3

.

**Energisation**,** control and detection of interconversions between Fe-S clusters.** In the standard case, a one-electron transfer in which both oxidised and reduced products are stable on the timescale of the experiment produces a simple signature of each active site. The voltammetry can be used to study how a redox-active site undergoes a relatively slow change in structure (Fig. 4A), an example being where a metal ion is added to or removed from a [3Fe-4S] cluster. Referring back to Fig. 2, the new signal due to the [Zn3Fe-4S]^2+/+^ couple grows in as the signals due to the [3Fe-4S] cluster ([3Fe-4S]^+/0^ and [3Fe-4S]^0/2−^, see later) disappear [1]. The reaction was easily reversed by transferring the electrode to a Zn^2+^-free solution and switching to an oxidising potential to release Zn^2+^. Since only a tiny quantity (pmoles/cm^2^) of protein is present on the electrode, the equilibrium constants for Zn^2+^ and other metal ions reacting with [3Fe-4S] clusters in different proteins could be measured even with micromolar-level metal ion concentrations in solution [1, 37, 38]. Incorporation of Zn^2+^ and other M^2+^ occurs when the [3Fe-4S]^+^ cluster is reduced by one electron, whereas for M^+^, entry occurs also (but weakly) at the 1+ level [2, 3]. For Tl^+^ the exchange is so fast that a simple voltammogram is observed and equilibrium constants are extracted from the shift in potential *E*_diag_ (Fig. 3) with Tl^+^ concentration [2]. Graduate students Raul Camba and Gareth Tilley addressed the common observation that [4Fe-4S] clusters are prone to oxidative damage. The first stage – formation of [3Fe-4S]^+^ via a transient *super-oxidised* [4Fe-4S]^3+^ species – could be studied easily by employing short oxidative pulses of the electrode potential [39].

**Redox-dependent ligand exchange – an electron pump.** An example of a cyclic stepwise **ECEC** process is the release and rebinding of an exogenous thiolate ligand by a [4Fe-4S] cluster having a potentially vacant coordination site (Fig. 4B). Such a process was studied for the labile [4Fe-4S] cluster in Ferredoxin III from *Desulfovibrio africanus* [40]. Proceeding clockwise round the cycle, reduction causes the thiolate ligand to dissociate, producing a stable state with a higher reduction potential: electron-transfer thus drives a ligand replacement reaction. It is easy to see that an anticlockwise process, in which the **C** steps are forced to run in reverse (as via an energised conformational change) would convert a mild reductant into a more powerful electron donor – an electron pump and a further example of energy coupling. The P-cluster of nitrogenase, which adopts different structures depending on redox state, comes to mind [41].

**Electron transfer gated by proton transfer.** Working with Barbara Burgess, my group studied a ‘model’ proton-gated electron transfer reaction. Reduction and re-oxidation of the reduced, protonated [3Fe-4S]^0^ cluster in Ferredoxin I from *Azotobacter vinelandii* (*Av* FdI) corresponds to the non-cyclic **ECCE** process shown in Fig. 3 [42]. In this case, species O’ (which would correspond to the protonated, oxidised cluster) is too unstable to exist, and the only pathway back to O is via R. The cluster is buried beneath the protein surface so the sequence represents a proton pump in one direction (**EC**) and a proton gate in the other (**CE**). A variant of *Av* FdI in which an aspartic acid residue located between cluster and solvent-exposed surface is replaced by asparagine (slowing down proton transfer) allowed coupling and decoupling to be resolved [42, 43]. Later, as a graduate student at Oxford, Judy Hirst applied trumpet plot analysis (Fig. 1C) to explore and quantify the time-dependent coupling across scan rates up to 100 V s^− 1^ [44]. Conditions of pH and scan rates were identified for which the reduction process consists of electron transfer followed by spontaneous H^+^ transfer (sharp peak) to give R’, whereas the return oxidation process, yielding O, is controlled by the (slow) rate of deprotonation that is limited by the ability of the carboxylate (or lack of it) to assist H^+^ transfer to bulk solvent (Fig. 4C). Consequently, at an appropriate scan rate, the oxidation peak is suppressed and flattened out. Accordingly, a rapid cycle commenced at the reducing limit showed very little oxidation current, whereas the return reduction process appeared as a peak because enough time has elapsed to regenerate the oxidised state. The rate constants of the electron and proton transfer steps for native and variant forms of FdI, interpreted alongside complementary x-ray structures and molecular dynamics calculations, led us to propose a ‘swinging arm’ mechanism for H^+^ transfer [45].

**[3Fe-4S] clusters can form a protonated all-Fe(II) state.** Numerous studies with different proteins revealed that [3Fe-4S] clusters have much richer redox chemistry than was apparent from conventional methods. In PFE, they display a characteristic electron-exchange signature, consisting of a one-electron signal corresponding to the well-known [3Fe-4S]^+/0^ couple and a sharp and strongly pH-dependent cooperative two-electron signal at around −700 mV (pH 6) assigned to the [3Fe-4S]^0/2−^couple (Fig. 4D) [46–48]. The straightforward explanation was that the hyper-reduced species is an all–Fe(II) state stabilised by protonation of the µ^2^-sulfido ligands, yet the cooperativity and possible concertedness in terms of the tightly coupled 2H^+^/2e^−^ transfer (making it difficult to generate using solution-based reductants) was puzzling. Judy Hirst and Guy Jameson, then a 4th year undergraduate researcher, found that the electron-exchange signal is very sensitive to H_2_O/D_2_O exchange – trumpet plot analysis suggesting a central role for cooperative two-proton transfer and the possibility of an intermediate having a S–S bond [48]. A physiological role has yet to be established, but the chemistry has fundamental relevance as proposed mechanisms for nitrogenase involve successive protonation of the µ^2^-sulfido ligands of FeMoco as the cluster is reduced [49, 50].

**Imidazole protonation in Rieske [2Fe-2S] centres.** The electrode carrying the protein molecules can be transferred between different solutions, allowing the properties of a redox-active site to be observed across a very wide range of environments, including hostile conditions that would make normal measurements impossible. Reduction potentials of so-called Rieske [2Fe-2S] centres (in which the two Fe atoms are coordinated by two cysteine-S and two histidine-N) span a range of at least 0.5 V at pH 7 ( > +0.35 V to < -0.15 V) depending on source [51]. By making measurements between pH 3 and 14, Hirst and co-workers obtained complete redox profiles (Pourbaix diagrams) for various Rieske [2Fe-2S] centres and concluded that the reduction potential is controlled by the (rapidly established) protonation state of the imidazole group: the high-potential centres are coordinated by neutral imidazole whereas the low-potential centres are coordinated by anionic imidazolate (His-imid^−^ being electrostatically equivalent to Cys-S^−^) [51, 52].

Some general lessons from these studies were as follows.

- Intensification of an electron-exchange signal due to two-electron cooperativity is very informative, not least because it can reveal a redox centre in a protein for which low electrode coverage would otherwise make detection very difficult. For enzymes, the electron-exchange signals obtained (rarely) in the absence of the substrate are known as non-turnover signals. More likely to be visible are flavin cofactors, as these commonly undergo highly cooperative two-electron transfer. In certain enzymes, cooperative two-electron centres carry out electron *bifurcation*, whereby the component one-electron transfers that are highly inverted in potential (*E*_2_ >> *E*_1_) are constrained to occur in separate steps and directions (Fig. 3). A one-electron transfer reaction that is otherwise prohibitively uphill thermodynamically is therefore driven by coupling to the favourable transfer of the other electron – exquisite coupling within organised multi-domain systems usually being necessary to ensure that this happens [53–58]. Bifurcation is difficult to resolve, but Minteer and Miller have recently shown how this can be achieved through PFE experiments with a bifurcating electron-transfer flavoprotein [59].
- Strangely, electrochemical methods were largely dismissed in earlier prominent literature dealing with gated electron transfer, an issue being ‘difficulties involving treatment of mass transport’ [60]. On the contrary, gated ET would prove to be easily diagnosed by PFE, which avoids diffusion of redox-active species and sharpens the response.
- By enabling the redox properties of an active site to be measured rapidly under unstable and non-physiological conditions, it is possible to determine the underlying basis of properties that cannot be understood without extrapolation. Aside from the Rieske centre experiments mentioned above, Sean Elliott and co-workers have used PFE to derive wide-range Pourbaix (*E* vs. pH) diagrams for thioredoxins that perform two-electron redox chemistry through CysS-SCys bond formation [61].

### The coupling of electron transfer in enzyme catalysis

I was introduced to metalloenzymes by Peter Kroneck, with whom I spent a transformative year as a Royal Society European Exchange Fellow at the University of Konstanz– my first postdoctoral stint after completing my PhD in 1978 with the legendary kineticist Geoff Sykes. Peter’s intellect and objectivity combined with his wit and enthusiasm would inspire me to make a career choice that I have never regretted.

The idea of exploiting immobilised enzymes as electrocatalysts was already alive by the 1970s through efforts by Soviet electrochemists [62–64]. Using thoroughly electrochemical language, the researchers addressed overpotential and directed attention to the relationship between turnover rate (frequency) and current. Since these beginnings, the applications of enzymes as electrocatalysts for biosensors and specialised fuel cells have been important drivers of research for over 40 years, led by prominent scientists such as Arkady Karyakin, Frieder Scheller, Lo Gorton, Allen Hill, Itamar Willner, George Wilson, Philip Bartlett, Adam Heller and many others [64–77]. With progress in innovating devices typically described in terms of 1st generation devices, 2nd generation… and so on, the mission-driven emphasis has been on achieving increasingly stable and selective responses to reactants that a particular enzyme acts upon, for example glucose with glucose oxidase: devices would even be designed to operate within living tissue [78, 79]. Although enzyme electrocatalysis has employed both direct and indirect (mediated) electron transfer, the latter has led the way in applications – understandably, since although an enzyme such as glucose oxidase does not function through long-range electron tunnelling, its active site is accessible to small electroactive molecules such as ferrocenes [69].

Yet it should come as no surprise that even giant electron-transport enzymes should undergo direct interfacial ET if appropriate conditions are met – most obviously, their biological function requires fast, long-range electron tunneling across extensive surface-surface (protein) contacts, often at membranes. The tunnelling distances involved became obvious in 3D structures from the 1960s onwards, and systematic examinations of how long-range ET into and within enzymes depends on distance and the intervening medium have since occupied many experts [8–11].

My group has focused on enzymes that are engaged through direct interfacial ET that is neither aided nor masked by electron mediators. Research has been aimed primarily at how PFE can yield detailed fundamental chemical insight, unravel kinetic complexity and reveal hitherto undetected electronic-like behaviour – an aspect which has turned out to be a common property of multi-centred electron-transport enzymes, yet one that is obscured in conventional approaches that fail to interrogate enzymes in action. In what I have sometimes called the ‘Martian’ analogy I have likened the use of PFE to examining the debris of a crashed unmanned alien spacecraft: to find out what a particular strange component did, we would wire it up to an electrical analyzer. A PFE experiment ‘wires up’ an enzyme to record and diagnose its characteristics.

In PFE, the electrode potential not only drives catalytic ET but it also acts to influence where electrons reside within the enzyme. A continuous scan across a wide potential range can identify irregular modulations due to advantageous or disadvantageous changes to the redox status of particular sites. Once asked by a reviewer why cyclic voltammetry rather than a single linear potential sweep is used (given that there should be no difference if a steady state is maintained) my answer was that only by cycling is it easy to spot hysteresis arising from slow potential-dependent activation or inactivation. Instability can even be an advantage, as the decaying currents of a reversible electrocatalyst trace out the formal potential as an isosbestic reference point.

Numerous enzymes are now known to display direct electron exchange and native activity when attached to a suitable electrode. Whereas many small proteins displayed conspicuous requirements for co-adsorbates, such as multivalent cations, larger proteins appeared less dependent on charge compensation, although it would be essential that a productive orientation is easily attained. In reality, (a) protein adsorption in which the native conformation is preserved depends on there being a suitable balance of hydrophobic effects (displacement of many water molecules) and polar (surface charge) interactions [80, 81]; (b) the interaction of a native enzyme with an electrode surface allows dynamic fluctuations (cartoons such as shown in Fig. 1A should not imply a static arrangement) and interfacial electron transfer occurs across a small, dispersed range of orientation and distance [82]. With the enzyme anchored, the electrode could be rotated at variable high speed to control the mass transport of reactants (inbound, minimising depletion) and products (outbound): this aspect would prove to be valuable. An anaerobic glovebox proved to be essential in cases where even traces of atmospheric O_2_ could interfere.

Referring back to Fig. 1B, if a redox centre responsible for the peak-like signal shown is also a catalyst, then addition of its substrate results in a catalytic wave in which the current may be greatly amplified. In practice, in the absence of turnover conditions, the redox sites of ET enzymes are rarely revealed as discrete peaks as found with small electron-transfer proteins: the coverage is usually too low to measure (although this limitation can be addressed with Fourier transform voltammetry [19, 20]). However, if non-turnover signals are observable when the reacting substrate is absent, kinetic information can be extracted from the scan-rate dependence of the catalytic voltammetry when the substrate is added: above a certain scan rate, catalytic action is ‘timed out’, restoring the non-turnover signal. As already mentioned, cooperative two-electron processes (Fig. 3) give much sharper signals. An interesting case arose with cytochrome c peroxidase, which displays a prominent non-turnover signal close to the onset potential for H_2_O_2_ reduction. From studies with native enzyme carried out by postdoc Madhu Mondal and 4th year undergraduate researcher Helen Fuller, followed up through a collaboration with Dave Goodin (then at Scripps)  using site-directed variants (leading in turn to further investigations by graduate student Libei Bateman) and further analysis by Fourier transform voltammetry, it was established that the two one-electron transfers to separate sites, heme-Fe and tryptophan, with *E*_2_ ~ *E*_1_, are tightly coupled [18, 19, 83, 84].

**Fig. 5:**
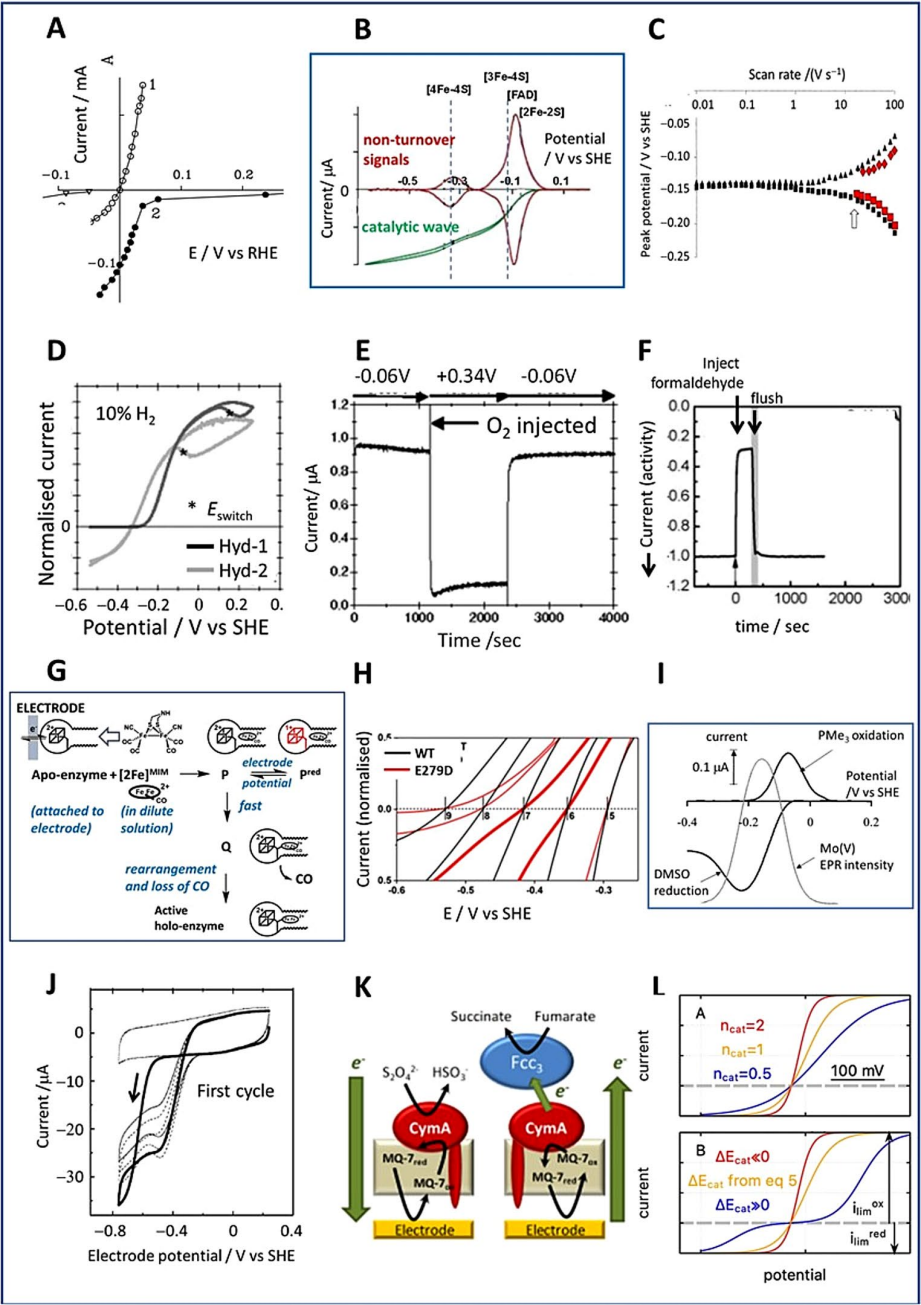
Images depicting different aspects of PFE applied to enzymes. **A** Polarisation curve (a point-by-point construction) for H^+^/H_2_ interconversion by a hydrogenase, probably the earliest published demonstration of reversible electrocatalysis by an enzyme (reprinted with permission, Elsevier, original kindly supplied by Professor Karyakin) [64]. **B** Catalytic voltammogram of *E. coli* fumarate reductase (green trace) overlaid on non-turnover signals of redox centres (red trace) [92]. **C** Trumpet plot for the FAD electron-exchange signal of Fcc_3_ – with (red symbols) and without (black symbols) fumarate present [93]. **D** Comparing anaerobic H^+^/H_2_ interconversion by two *E.coli* hydrogenases, Hyd-1 and Hyd-2 [94]. **E** Chronoamperometry of O_2_-tolerant Hyd-1 showing response to O_2_ exposure [94]. **F** Chronoamperometry showing the inhibition by formaldehyde of a [FeFe]-hydrogenase catalysing H_2_ production [95]. **G** Scheme showing how final assembly of the H-cluster depends on the valence-electron population of the product [96]. **H** Deviation from electrocatalytic reversibility due to minor disruption of the H^+^-transfer pathway in a [FeFe]-hydrogenase: results are for different pH values [97]. **I** DMSO reduction and PMe_3_ oxidation catalysed by DMSO reductase, overlaid on the appearance of Mo(V) EPR signal [98]. **J** How reductive activation of a nitrate reductase appears as hysteresis (reprinted with permission, Royal Society of Chemistry) [99]. **K** Electrodes modified with scaffolded membranes to host enzymes [28, 86]. **L** Modeling electrocatalysis by enzymes [85]

Some images of the many different aspects of PFE are shown in Fig. 5. Several reviews on PFE expand on these and other aspects [85–91].

.

**Fig. 6:**
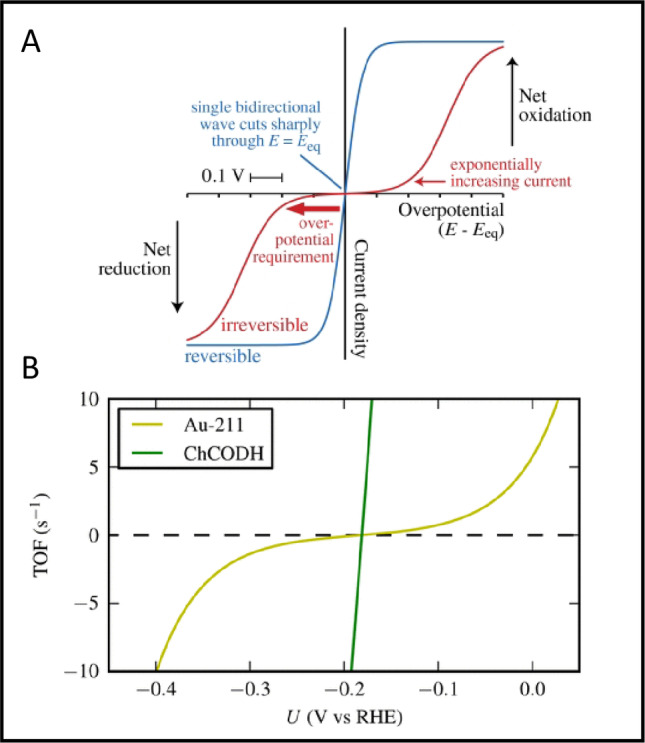
**A** Reversible (blue) versus irreversible (red) electrocatalysis. The opposing scenarios refer to a situation in which both oxidised and reduced forms of the redox couple are present in solution [100]. **B** Comparing catalytic CO_2_/CO interconversion by a metal electrode and an enzyme [104]

**Efficiency and reversibility in electrocatalysis by enzymes.** As now established for many enzymes – if both oxidised and reduced forms of the reactant are present, the catalytic wave intersects the zero-current axis sharply at the expected equilibrium potential, the immediate increase either side signifying that the electrocatalytic process is reversible (Fig. 6A) [100]. Electrocatalytic reversibility, first demonstrated for a hydrogenase (Fig. 5A), was otherwise reserved for platinum and the reversible hydrogen electrode [64]. Reversibility, in the strict electrochemical sense, must be distinguished from bidirectionality, the latter describing a reaction that can trivially be driven in either direction, rather than one for which the reaction rate and direction respond to the smallest change in potential at or close to the reversible potential – i.e. there is minimal overpotential requirement, indicative of a low activation barrier [100–103].

Electrocatalytic reversibility is an intriguing metric for bio-inspired redox catalysis and merits attention alongside more conventional concepts relating to the extraordinary catalytic performance of enzymes. During the 1970s, Albery and Knowles gauged enzyme efficiency in terms of how closely the catalysed reaction rate approached diffusion control [105]. In 2011, a paper by Judy Hirst and myself addressed the efficiency of electron-transferring enzymes in ways familiar to inorganic chemists (Pourbaix and Frost diagrams), highlighting how overpotential requirements are minimised, and advocating enzymes as benchmarks for electrocatalysts [100]. An interesting hypothesis is that the need to minimise overpotential would have been a driver in early evolution, adding emphasis to earlier (and continuing) ideas concerning the electrochemical nature of life [91, 106–108]. The significance of enzyme electrocatalytic reversibility and the stark contrast with industrially important electrocatalysis was quickly recognised by Nørskov and colleagues in a paper on electrocatalytic CO_2_ reduction [104]. The authors compared the irreversible electrocatalysis displayed by a Au electrode with that of a Ni-containing carbon monoxide dehydrogenase attached to PGE [109]: the voltammogram of the enzyme displays a steep cut through the zero-current axis (Fig. 6B).

**Fig. 7:**
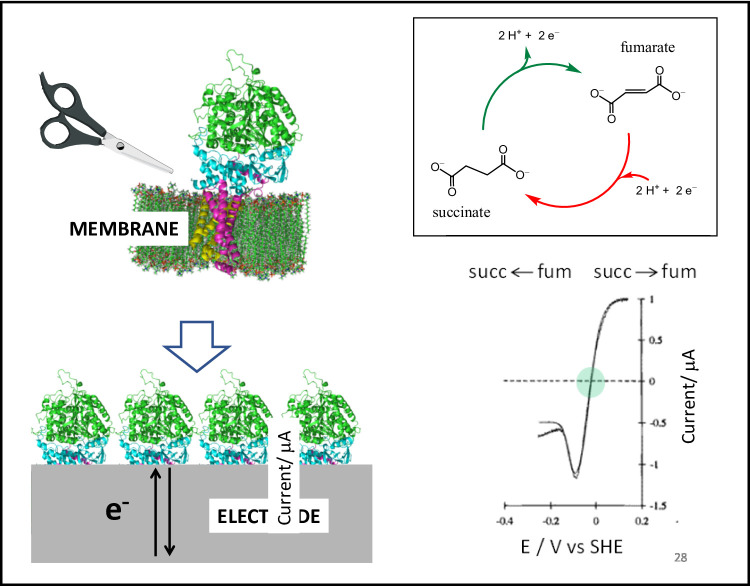
Concept and early results showing reversible electrocatalysis by the membrane-extrinsic domain of succinate dehydrogenase (mitochondrial Complex II) [110]. Metaphorically, the membrane is swapped for an electrode

**Multicentred flavoenzymes.** Succinate dehydrogenase (SDH) which exists in membrane-bound form as succinate-ubiquinone oxidoreductase (Complex II in mitochondria) is an enzyme of the TCA cycle. Working with Brian Ackrell who was based at the VA Medical Center in San Francisco, postdoc Artur Sucheta in my group at UCI made an interesting observation. In a metaphoric sense, the electrochemical procedure involved the membrane anchor peptides of the intact enzyme being replaced by a PGE electrode (Fig. 7) [110].

The electrode would thus function like a continuously adjustable quinone pool and report on how the enzyme, which contains an active-site FAD and a relay formed of three Fe-S clusters, responds to the changes in potential. The direction of interconversion between fumarate and succinate (each present in solution at equal concentration) switched sharply at the expected formal potential (green oval), thus demonstrating that the enzyme functions as a reversible electrocatalyst [110]. We observed that the current due to fumarate reduction *decreased* sharply as the overpotential is increased beyond a certain threshold: the enzyme was thus behaving like a tunnel diode, an electronic device displaying *negative resistance*. After I moved from California to Oxford, the discovery was analysed in more detail by Judy Hirst and later by Harsh Pershad [111]. The property was also observed in a bacterial SDH and reproduced in conventional measurements [112–115]. Various physiological implications have since been associated with the tunnel diode effect, which is a consequence of the electrode potential favouring a less active state of the enzyme, in this case under more reducing conditions. Some unusual experiments exploited the ability to make simple repetitive exchanges of the electrolyte solution (D_2_O for H_2_O and deuterated vs. undeuterated substrates). Here, PFE provided a unique opportunity to quantify H/D isotope substitutions on the different aspects of catalysis by SDH in a very rigorous way, with kinetic and thermodynamic influences being automatically calibrated and easily deconvoluted [116].

Teaming up with Joel Weiner and Gary Cecchini, we investigated an analogous enzyme, fumarate reductase (FRD) from *E. coli*, which like SDH has a covalently bound FAD as the site of catalytic hydride transfer to fumarate [117, 118]. As with other electron-transferring flavoenzymes, the FAD links a two-electron interconversion to sequential long-range one-electron transfers. No such tunnel diode effect was observed, but FRD not only also behaved as a reversible electrocatalyst, but adsorbed to high coverage, thus allowing its active sites to be observed as non-turnover signals in the absence of fumarate or succinate (Fig. 5B) [92]. Graduate student Janette Hudson estabished that the catalytic wave, which commences at the potential of the envelope encompassing FAD, [2Fe-2S] and [3Fe-4S] centres, displayed a boost at the potential of the [4Fe-4S] centre located midway along the relay. Non-turnover signals were also observed with another fumarate reductase, flavocytochrome *c*_3_ (Fcc_3_) from a marine bacterium *Shewanella fridigimarina*, in investigations made in collaboration with Stephen Chapman at the University of Edinburgh. As with FRD, the prominent two-electron signal from the FAD cofactor was clearly observable for Fcc_3_ above an envelope of overlapping signals from the four heme groups that comprise the electron relay [119]. Using trumpet-plot analysis (Fig. 1C), Anne Jones, then a graduate student in my group, obtained a rare glimpse of the FAD in Fcc_3 _*in action* during intramolecular ET and catalytic turnover [93]. In the presence of fumarate, the smaller separation of the FAD electron-exchange peaks (arrowed) appearing upon collapse of the catalytic wave at 14 V s^− 1^ indicated that ET is faster in the presence of the substrate (Fig. 5C). Extraction of the parameters *k*_cat_ = 120 s^− 1^ and *k*_0_ = 500 s^− 1^ represented an early example of the deconvolution of a steady-state enzyme electrocatalytic process. With Lars Jeuken, Anne applied square-wave voltammetry to compare the electron relay characteristics of both fumarate reductases, acquiring evidence for heme-heme super exchange in the case of Fcc_3_ and rate-limiting internal electron transfer for *E. coli* FRD [120].

**Hydrogenases**. Hydrogenases became inspirational in the growing green energy field after the structures of [NiFe]- and [FeFe]- enzymes were solved in the mid-1990s [121–123]. It had already been reported that a [NiFe]-hydrogenase behaves as a reversible electrocatalyst for H^+^/H_2_ interconversion [64], and interest in their electrochemistry began to expand during the 1990s [124, 125]. These enzymes, which are normally very sensitive to O_2_, display great complexity in their behaviour, and detailed investigations by PFE helped resolve many issues, including the origins of O_2_ tolerance across the different classes and sub-classes. The direct relationship between catalytic turnover rate and current meant that changes in catalytic rate caused by any interruption (addition/removal of any reagent, or change in potential) were immediately detected and quantified (rather than taking the derivative of a product/time slope), thereby facilitating trailblazing and detailed investigations.

In Oxford, hydrogenase research began in 1995 after I had discussed with Siem (aka Simon) Albracht of the University of Amsterdam the possibility of using PFE to help resolve the intricate electrocatalytic properties of the [NiFe]-hydrogenase from *Allochromatium vinosum*. The first studies were carried out by graduate students Jill Duff and Harsh Pershad [126]. Now that the structure of a closely related [NiFe]--hydrogenase had been solved and the redox properties of the different electron-relaying Fe-S centres could be correlated, the catalytic voltammetry combined with non-turnover signals from the Fe-S centres (obtained upon inhibition by CO) proved that H^+^ reduction (H_2_ evolution) occurred rapidly despite the medial [3Fe-4S] centre having a reduction potential > 0.3 V more positive than *E*_eq_. It is worth noting that for any hydrogenase competent in H_2_ production, equilibrium (mid-point) potentials obtained by conventional EPR titrations across and below the region of the reversible H^+^/H_2_ potential must be treated with scepticism, as the enzyme would need to be inactive to permit such measurements. From 2001, PFE would play a central role in trailblazing, testing and predicting many features of enzymatic H_2_ activation, each navigated early on through productive collaborations and some outstanding researchers in my group. Postdocs Christophe Léger and Kylie Vincent along with graduate students Anne Jones, Sophie Lamle, Alison Parkin, James Cracknell, Gabrielle Goldet and Natalie Belsey were first in developing extensive and intricate protocols to investigate the electrochemical kinetics of H_2_ activation and the potential- and O_2_-dependent interconversions between different states [82, 127, 128]. We used cyclic voltammetry and chronoamperometry – carrying out detailed studies made possible by use of a rotating disc electrode fitted into a sealed cell in which the headspace gas composition was regulated by a flow mixer [129]. In collaboration with Juan Fontecilla-Camps at CNRS in Grenoble, Alison and Gabrielle carried out the first investigations of a [NiFeSe]-hydrogenase, which is far more active for H_2_ production than standard [NiFe]-hydrogenases [130].

In 2004 we started collaborating with Baerbel Friedrich at the Humboldt University, Berlin, along with Oliver Lenz who would later move to the Technical University, to help unravel the special properties of the O_2_-tolerant [NiFe]-hydrogenases discovered in *Ralstonia eutropha (Cupriavidus necatur)* [131–136]. Over the following years, the ‘[NiFe]- team’ in Oxford would include graduate students Maxie Roessler, Michael Lukey, Annemarie Wait, Günter Knüdelhäuser, Bonnie Murphy and Kourosh Ebrahimi. Frank Sargent, then at the University of Dundee, encouraged us to work on the three [NiFe]-hydrogenases expressed by *E. coli.* We made careful comparisons of *E.coli* Hyd-1 (which is O_2_-tolerant) and Hyd-2 (which is a standard O_2_-sensitive enzyme and proficient at H_2_ production) [137]. Bonnie Murphy also studied Hyd-3 which is part of the formate hydrogenlyase complex [138]. The activities of Hyd-1 and Hyd-2 under anaerobic conditions are compared in Fig. 5D. Hyd-2 behaves as a reversible electrocatalyst, whereas Hyd-1 does not catalyse H_2_ production and requires a sizeable overpotential for H_2_ oxidation. As observed earlier with other [NiFe]-hydrogenases, at a sufficiently positive potential, both enzymes revert to an inactive state – a Ni(III)-OH complex corresponding to the EPR-characterised species called Ni-B – but they undergo rapid reductive re-activation as the electrode potential is returned in the negative direction [139]. Graduate student Suzannah Hexter and postdoc Thomas Esterle would later draw comparisons with catalysis by Pt and Ru, each of which form a passive oxide layer at sufficiently positive potential [140].

It was becoming clear that O_2_-tolerance is associated not only with restricting the physical access of O_2_ to the active site, but also with the presence of an unusual proximal [4Fe-3S] cluster that is linked to the protein by six cysteines [141, 142]. This centre is able to undergo two sequential one-electron transfers, through a rearrangement that is coupled to removal of the second electron [143, 144]. Maxie Roessler worked alongside Jeffrey Harmer to investigate the special cluster using pulse EPR methods [145]. In 2010, postdoc Rhiannon Evans joined the group to spearhead systematic investigations of Hyd-1 and Hyd-2 using site-directed mutagenesis [106, 146–148]. Rather than relying on simple injections of O_2_, which would rapidly escape and reveal only short-lived effects, it was important to examine the potential and time dependences of exposure to O_2_ for extended periods (Fig. 5E). We proposed that an important role of the unusual proximal [4Fe-3S] cluster lay in ensuring an adequate supply of ‘rescue’ electrons to the [NiFe] active site as it is attacked by O_2_, thus preventing attack by reactive O-intermediates [148]. The product, Ni-B (‘Ready’), is quickly reactivated by electron transfer and H_2_, whereas the damaged oxygenated product known as Ni-A (‘Unready’) is not. Ultimately, graduate student Philip Wulff carried out ^18^O_2_ mass spectrometry experiments to establish that O_2_-tolerant [NiFe]-hydrogenases rescue themselves through their ability to function as 4-electron oxidases [149]. Through further close collaborations with Simon Phillips and Stephen Carr (Diamond Light Source), Michael Haumann and Ingo Zebger (Berlin), and Kylie Vincent and Will Myers in Oxford, we added detailed X-ray structural and spectroscopic studies to the repertoire. Meanwhile, Rhiannon Evans, undergraduate researcher Sara Wehlin and graduate students Tania Islam and Stephen Beaton pushed ahead with the molecular biology. An as-yet unresolved outcome of this work was our proposal that the H_2_ molecule is immediately cleaved (or formed) via a ‘frustrated Lewis pair’ reaction using the guanidinium (transiently guanidine) side chain of a conserved arginine lying just above the Ni and Fe atoms. The R509K variant of Hyd-1, in which the pendant arginine is replaced by lysine displayed a very large attenuation of electrocatalytic activity [150–152].

We were also investigating [FeFe]-hydrogenases in two collaborations, first with Juan Fontecilla-Camps, then later with Thomas Happe and Sven Stripp of the Ruhr University, Bochum, with whom we studied the enzymes from *Clostridium pasteurianum*, and *Chlamydomonas reinhardtii*, the latter lacking an Fe-S relay [153–155]. Although [FeFe]-hydrogenases are extremely active, they are highly O_2_-sensitive, and by exploiting the ability of PFE to control the electrode potential (allowing states of interest to be ‘pinned’) we investigated their reactions with O_2_ [156]. It would later be discovered that sulfide protects [FeFe]-hydrogenases from O_2_ by binding to the target Fe in the 2Fe component of the H-cluster [157].

Our progress on [FeFe]-hydrogenases involved some unusual experiments, summarised as follows:

- Following trailblazing investigations by graduate student Carina Foster and 4th year undergraduate researcher Caterina Brandmayr, we established that formaldehyde, a strong electrophile, is a strong inhibitor of H_2_ evolution by [FeFe]-hydrogenases – activity being restored quickly (Fig. 5F) when the solution is exchanged for one free of aldehyde – whereas it is a poor and irreversible inactivator of H_2_ oxidation [95, 158]. Based on pulsed-EPR studies with ^13^C -labelled formaldehyde and DFT calculations, we proposed that formaldehyde reacts with the two-electron reduced H-cluster to give an adduct having a Fe-C bond [159]. Andreas Bachmeier carried out much of this work, including experiments with other aldehydes which showed decreasing inhibition with increasing chain length: his doctoral thesis was selected for publication by Springer in their ‘Recognising Outstanding Ph.D. Research’ series [160].
- Shortly after she joined as a postdoc, Clare Megarity exploited the ability of PFE to pin particular redox states of an enzyme to examine the ‘final stage’ of formation of the active site, historically known as the ‘H-cluster’. The question is hypothetical in biological terms, but it addressed an interesting electron-counting principle. As summarised in Fig. 5G, Clare found that fusion of the two components, the synthetic [2Fe] complex (contained in solution) and the [4Fe-4S] cluster (in apo-protein on the electrode) does not occur if the electrode potential is sufficiently negative that the product would be too electron-rich (requiring anti-bonding orbitals to be occupied) [96].
- Visiting researcher Kavita Pandey used impedance spectroscopy to study the frequency dependence of catalytic electron transfer by two [FeFe]-hydrogenases [161]. She was thus able to measure the ‘catalytic electron-transfer exchange rate’ – a unique metric, akin to the idle rate of a car engine – as the driving force tends to zero. The two hydrogenases displayed 2 H^+^/H_2_ exchange rates of 25 and 78 molecules H_2_ s^−1^, the lower value being for the enzyme lacking a Fe-S relay.

Hydrogenases are unique enzymes in acting upon the simplest and lightest of substrates, electrons, protons and molecular H_2_. The tight coupling between electron and proton transfers was clearly revealed in carefully-designed PFE studies on [FeFe]- and [NiFe]-hydrogenases. Experiments with [FeFe]-hydrogenases revealed the role of *remote* proton transfer. As a visiting student, Oliver Lampret from Thomas Happe’s group used PFE to study variants of [FeFe]-hydrogenases from *Clostridium pasteurianum* and *Chlamydomonas reinhardtii*, each of which had a single residue replaced (Glu to Asp) along its proposed proton-transfer pathway [97]. With the *C. pasteurianum* enzyme, the proton-transfer pathway lies far away and opposite the electron relay. The replacements resulted in only partial loss of catalytic activity when measured conventionally, so that they would still be active for PFE. Both [FeFe]-hydrogenases displayed a clearly visible inflection in their electrocatalytic voltammetry trace as the current switched direction at the reversible potential (Fig. 5H), signifying (with reference to Fig. 6A) the likely direct involvement of concerted PCET in achieving electrocatalytic reversibility.

Experiments in Oxford with [NiFe]-hydrogenases demonstrated the role of tightly-coupled *adjacent* proton transfer. Continuing as a postdoc, Bonnie Murphy was instrumental in setting up a new collaboration with Dieter Söll of Yale University, with whom we designed experiments to test the effects of systematic disruptions to the inner coordination shell around the Ni atom. Natalie Krahn, then a graduate student in Dieter’s lab, produced variants of *E. coli* [NiFe] Hyd-1 and Hyd-2 in which each individual cysteine-S ligand coordinating the active-site Ni was replaced by selenocysteine-Se [162, 163]. During extensive investigations by PFE, one variant stood out – Hyd 2 C546U, in which selenocysteine replaces cysteine-546, long known to lie in the pathway for proton transfer along the sequence NiH ↔ Cys-S ↔ Glu-COO^−^ ↔ solvent [164, 165]. Unlike Hyd-1, Hyd-2 is a reversible electrocatalyst, thereby allowing the crucial region around the equilibrium potential to be closely inspected. We found that whereas the electrocatalytic voltammogram of native Hyd-2 shows a clean cut through the zero-current axis at the reversible potential, the variant displayed an inflection, the change to a more sigmoidal trace signalling a breakdown in reversibility assignable to partial decoupling of concerted PCET. For comparison, no such inflection was observed with the pendant arginine-to-lysine variant (R479K) of Hyd-2, suggesting that proton-electron decoupling is not the reason for the large attenuation of activity observed in that case [152].

**Molybdenum-containing enzymes.** From 2000, we set about establishing how PFE could uncover useful new information on enzymes containing Mo-pterin cofactors, for which spectroscopic studies are largely limited to the intermediate Mo(V) state. Graduate student Kerensa Heffron was joined by postdocs Sean Elliott and Kevin Hoke, and we collaborated with John Enemark, Joel Weiner and Russ Hille. In two cases-  dimethyl sulfoxide (DMSO) reductase and nitrate reductase - the electrocatalytic voltammetry showed a potential optimum, analogous to the tunnel-diode effect found for SDH (Fig. 5I) [98, 166, 167]. Earlier, and independently, Julea Butt and her colleagues had obtained similar data for other nitrate reductases [98, 166]. Two explanations have been considered for this behaviour; (a) that substrates bind preferentially in the Mo(V) state, or (b) the pterin is redox active, thus complicating activity further. In their analysis of the unusual potential dependence, Léger and co-workers have emphasised how reduction potentials obtained by static potentiometry are useful only as a guide because they do not correspond to turnover conditions [168]. In another example, Butt and co-workers gave a particularly vivid demonstration of how cyclic voltammetry reveals reductive activation through hysteresis (Fig. 5J) [99]. Sulfite oxidase presented an example of an enzyme in which the electron-transferring centre (a heme group) and the Mo-pterin cofactor are located on separate domains, requiring a hinge-bending motion during the catalytic cycle [169]. The electrocatalytic oxidation of sulfite commenced at the potential of the heme group, which was also visible through its non-turnover signal (the Mo-cofactor not being detected). Another hinged-domain enzyme, cellobiose dehydrogenase (heme-flavin), has been extensively investigated by Gorton and colleagues [170–172]. Unusually, studies of arsenite oxidase revealed a narrow and prominent non-turnover signal which we assigned to a cooperative two-electron Mo(VI)/(IV) process, the inference being that the Mo(V) state has only a narrow potential window of stability in this enzyme [173].

**Enzymes catalysing CO**_**2**_**reduction.** Joining forces with Steve Ragsdale in 2006, we began investigating Ni-containing carbon monoxide dehydrogenases (Ni-CODH). The research, initiated originally by Alison Parkin then continued by graduate students Vincent Wang and Tania Islam revealed and clarified properties undetected by conventional methods, including that of electrocatalytic reversibility. Here it was necessary to control the levels of both CO and CO_2_. Vincent discovered that just as CN^−^ is a strong inhibitor of CO oxidation, NCO^−^ is a strong inhibitor of CO_2_ reduction [174, 175]. Holger Dobbek subsequently determined the crystal structure of cyanate-inhibited CODH which showed NCO^−^ coordinated to the Ni through the C atom and formulated as a two-electron reduced carbamoyl group: this had important mechanistic implications in view of evidence for a Ni-carbonite intermediate [176, 177]. The other major class of CO_2_-reducing enzymes are the W- and Mo- containing formate dehydrogenases. Judy Hirst in Cambridge, later also with Erwin Reisner, used PFE to make detailed studies of these enzymes, showing that like Ni-CODH they also function as reversible electrocatalysts [178, 179].

**Blue Cu oxidases.** Many researchers, including Harry Gray, Lo Gorton, Adam Heller and others, have used voltametric methods to study blue Cu oxidases, enzymes attracting widespread interest for their applications in biotechnology [180]. I had a special interest myself, as I had worked on ascorbate oxidase in Peter Kroneck’s group around the time that the structure of the Type 1 Cu centre, responsible for the intense blue colour, was being revealed in different proteins [181]. Certain blue Cu oxidases - bilirubin oxidase and fungal laccases - display just a small overpotential for the four-electron reduction of O_2_ under neutral/weakly acidic conditions [182]. It was well known that the oxygen reduction reaction (ORR) and oxygen evolution reaction (OER) limit the efficiency of fuel cells and electrolysers, and since we were interested in the possibility of exploiting hydrogenases in niche fuel cells, we started our own investigations of the oxidases. These enzymes normally catalyse the oxidation of organic molecules that bind in a hydrophobic pocket close to the Type 1 Cu. The biological side was taken forward by Oxford plant scientist Sarah Gurr, along with postdoc Stephen Giddens and graduate student Caroline Rodgers.  In 2006, Chris Blanford, then a postdoc in my group, and graduate student Rachel Heath discovered that the electrocatalysis of O_2_ reduction by laccase was greatly improved, in terms of current and stability, if anthracene units were covalently attached ‘end-on’ to the PGE electrode surface. We reasoned that like locating pins, these groups insert into the Type 1 pocket to guide enzyme orientation and enhance electronic coupling [183]. With Victor Climent and visiting student Luciano dos Santos, we went on to make further instructive comparisons with the ORR at a Pt electrode [184].

Encouraged by the small overpotentials required by blue Cu oxidases for O_2_ reduction, we applied a basic model for catalytic bias we had derived in 2002 (see below) to predict conditions under which water oxidation should be observable, the expectation being that bilirubin oxidase could behave as a reversible electrocatalyst. Assuming that the Type 1 Cu serves as the electrochemical control centre and that its reduction potential remained constant with pH, we predicted the pH at which the O_2_/2H_2_O and Type 1 Cu(II)/(I) potentials would become equal. The electrochemistry was unstable at the expected ‘sweet spot’ of pH 10.2 but as the activity decayed it traced out an isosbestic point centred at 1.23 V vs. RHE (reversible hydrogen electrode) – unconventional but compelling evidence that the complex trinuclear Cu site connected to the Type 1 Cu functions transiently as a reversible electrocatalyst for the four-electron O_2_/2H_2_O interconversion [106].

**Following on and summing up.** Potential applications of hydrogenases were explored. For enzyme-fuel cells the major emphasis was on O_2_-tolerant [NiFe]-hydrogenases, and work extended to membrane-less fuel cells that could operate using non-explosive mixtures of hydrogen in air [134, 185]. Inspired by an article from Antonio De Lacey [186], Gopan Krishnan (then a postdoc) used electrodes in which the surface area was greatly increased by the attachment of multiwalled carbon nanotubes: this enabled much-improved performance of the fuel cells [187]. The research, continued by Lang Xu, produced results having some interesting fundamental relevance: we could generate power from a weak (4%) H_2_/air mixture, but the fuel cell was unstable as Hyd-1 reverted too easily to Ni-B when the voltage was dropped (as with short circuiting), thus requiring an electron as well as H_2_ to re-start [188, 189]. Later, an extremely O_2_-tolerant [NiFe]-hydrogenase was discovered in an organism able to survive on traces of H_2_ [190].

In his subsequent independent career, Lars Jeuken went on to devise electrodes having scaffolds to support membranes (capable of containing electroactive quinones) thus creating environments for enzymes that are closer to biological reality (Fig. 5K) [28, 86, 191]. A wider interpretation of PFE is that it extends to any system in which a protein undergoes electron exchange with an energizable non-biological material. In a simple example, two electron-transferring enzymes, one catalysing oxidation, the other reduction, can become electrically connected through co-attachment to a conducting chassis. We discovered that graphite flakes formed by abrading the surface of pyrolytic graphite could be loaded with a hydrogenase and a second ET enzyme to produce catalytic particles. In a colloidal suspension, the reaction of the second enzyme was coupled to hydrogen oxidation via fast intra-particle electron transfer [192, 193]. Two examples, nitrate reduction using a nitrate reductase as second enzyme, and the water-gas shift reaction using CODH as second enzyme, helped pave the way for the HydRegen technology mentioned below.

In similar vein and expanding the repertoire in another direction, once Erwin Reisner had joined my group in 2009, we started to test the ability of enzymes to operate as solar fuel catalysts when attached to semiconducting nanoparticles. Our goal was to see if enzymes, being so efficient, would offer new insight regarding the limits of performance of conventional solar-fuel devices [194–198]. Since moving to Cambridge in 2010, Erwin has taken great strides in developing novel technology for artificial photosynthesis, along with many concepts and solutions for achieving a carbon-neutral future [199, 200]. The research on enzyme-nanoparticle hybrids, continued in my group by graduate students Tom Woolerton, Sally Sheard and Andreas Bachmeier, together with visiting researcher Yatendra Chaudhary, took different directions in Oxford and elsewhere, particularly in the laboratory of Paul King [201–206]. In 2017, Liyun Zhang came to Oxford to investigate how [NiFe]-hydrogenases and CODH could be energised with light by attaching Ag quantum dots to surface-exposed cysteine residues, both natural and then introduced by site-directed mutagenesis [207–209].

Kylie Vincent, who had joined me as a postdoc in 2001 and became an independent researcher after 2007, would go on to lead a new direction – developing innovative spectroscopic tools that allowed detailed characterisation of enzymes immobilised on an electrode and undergoing catalytic turnover. Vibrational spectroscopy applied to electrochemically-controlled proteins, including hydrogenases in solution, was already an established area [165, 210]. Kylie and colleagues went a step further by inventing Protein Film Infra-Red Electrochemistry (PFIRE), a powerful spectroscopic technique for identifying different states of hydrogenases (and other enzymes, particularly those binding CO or CN^−^) attached to an electrode at which catalysis would be driven under strict potential control [211–214]. Before long they had extended PFIRE to the spectroelectrochemical investigation of enzymes in the crystalline state [215, 216]. Simultaneously controlling and observing the status of redox-active sites of electrode-confined enzymes performing prolonged periods of steady-state catalysis would henceforth become a recognised area of research, and by 2019 Erwin and his co-workers were already able to appraise the growing number of methods they and others were using [217].

The lessons learnt from these studies of enzymes as electrocatalysts are summarised as follows.

- PFE reveals oft-hidden properties of enzymes, aspects that either cannot be measured or even observed through conventional approaches: they include potential-dependent internal phenomena such as the tuned optimisation of steady-state redox level, and the potentials and rates of inactivation and activation processes.
- Catalytic bias – a preference for catalysing a reaction in one direction and a particular property of enzymes with bound redox cofactors – is easily measured. We proposed in 2002 that the primary bias is related to the separation between the formal reduction potential of the site at which electrons enter or leave the enzyme (we referred to this site as the ‘electrochemical control center’) and the formal potential of the reactant [218]. The hypothesis led to predictions, borne out by experiments, of the conditions required to observe bidirectional and reversible electrocatalysis by an O_2_-tolerant hydrogenase and a blue Cu oxidase [106, 219]. Catalytic bias also results from changes in the state of the enzyme that depend on the potential that is applied [140, 155]. Despite our attempts to find simple rules, the concept of catalytic bias is undoubtedly more complex than the simple ‘Oxford’ models suggest, and Christophe Léger and his colleagues have written inspiring papers that discuss the various underlying factors [220–222].
- Enzymes possessing extended electron relays operate effectively in each direction despite the internal centres not following a smooth trend in reduction potentials. These points became clear from the bidirectional and reversible electrocatalytic behaviour of [NiFe]-hydrogenases and the membrane-extrinsic domain of Complex I (NADH: quinone oxidoreductase) [126, 223]. The relay formed from seven Fe-S clusters in Complex I provides an excellent example of the ‘roller-coaster’ model [11, 224, 225] and an informative account of the implications for efficiency (Complex I is a proton pump) has been written by Judy Hirst and Maxie Roessler [226].
- Numerous redox enzymes are now known to function as reversible electrocatalysts.
- Minimising overpotential may have been a driver in the early evolution of lower organisms faced with extracting energy from a limited thermodynamic range.
- Concerted PCET plays an important role in underpinning electrocatalytic reversibility.
- Oxidation and reduction do not need to share the same mechanism. As drawn, catalytic cycles misleadingly imply microscopic reversibility; but in the case of electrocatalysis (apart from catalysis at or very close to the reversible potential) net oxidation and reduction are often driven irreversibly at large overpotentials, conditions under which different states of active sites probably prevail.
- Important insight on intramolecular ET in enzymes arises from analysis and modelling of catalytic waveforms. An early generic example from Judy Hirst and Dirk Heering in my group [227] initiated ideas and opinions featuring a variety of views and angles, many stemming from Christophe Léger and co-workers (Fig. 5L). Outputs have ranged from dispersion (the spread of interfacial tunnelling rate constants due to variability in local orientation) to the complex state-dependent couplings occurring within ET relays [82, 85, 89, 222, 228, 229].
- The electrochemical metrics for enzyme catalysis provide useful inspiration and benchmarks for small molecular electrocatalysts – one only needs to see the many creative examples proposed and demonstrated by Andy Borovik, Wendy Shaw, Jenny Yang, Vincent Artero and others [230–235]. A frequent question about enzymes runs along the lines ‘how much of the enzyme could be trimmed away and still leave a functional core ?’ The analogous questions posed to coordination chemists are ‘what kind of outer-shell structure needs to be built onto the basic complex ?’ Evolution appears to have perfected the catalysts that can be formed from 21 amino acids and a few metallic elements. Important components relate to the coupling of electron and proton transfers and applying the ‘frustrated Lewis pair’ concept introduced by Doug Stephan and Gerhard Erker [236]. Designing bioinspired catalysts requires that we copy Biology’s strategies, not necessarily its structures [100].
- When hydrogenases and CODH were incorporated into devices for solar fuel production, the motivation was that the efficiency of such catalysts would be reflected in excellent performance under visible light. While this expectation proved to be the case, it became clear that the high cost, large electrode footprint and poor stability of enzymes would ultimately limit their applications to those leading to a much higher value product. In lectures, I have sometimes likened enzymes to Formula-1 racing cars – vehicles having very high performance but poor durability, so they require numerous pit-stops to replace worn parts. A better option, I suggested, would be that enzymes should be useful in applications likened to ‘pop-up shops’ – business environments in which only a day’s activity is required. A company, HydRegen, spun out by Kylie Vincent and Holly Reeve, in which efficient hydrogen-water interconversion is used to drive enzyme-based organic transformations, is testimony to the feasibility of commercial exploitation of the electrochemistry of hydrogenases [237–239]. As described next, the e-Leaf offers an unusual platform for enzyme science and technology that may lead to interesting new applications.

## The electrochemical leaf (e-Leaf)

**Fig. 8:**
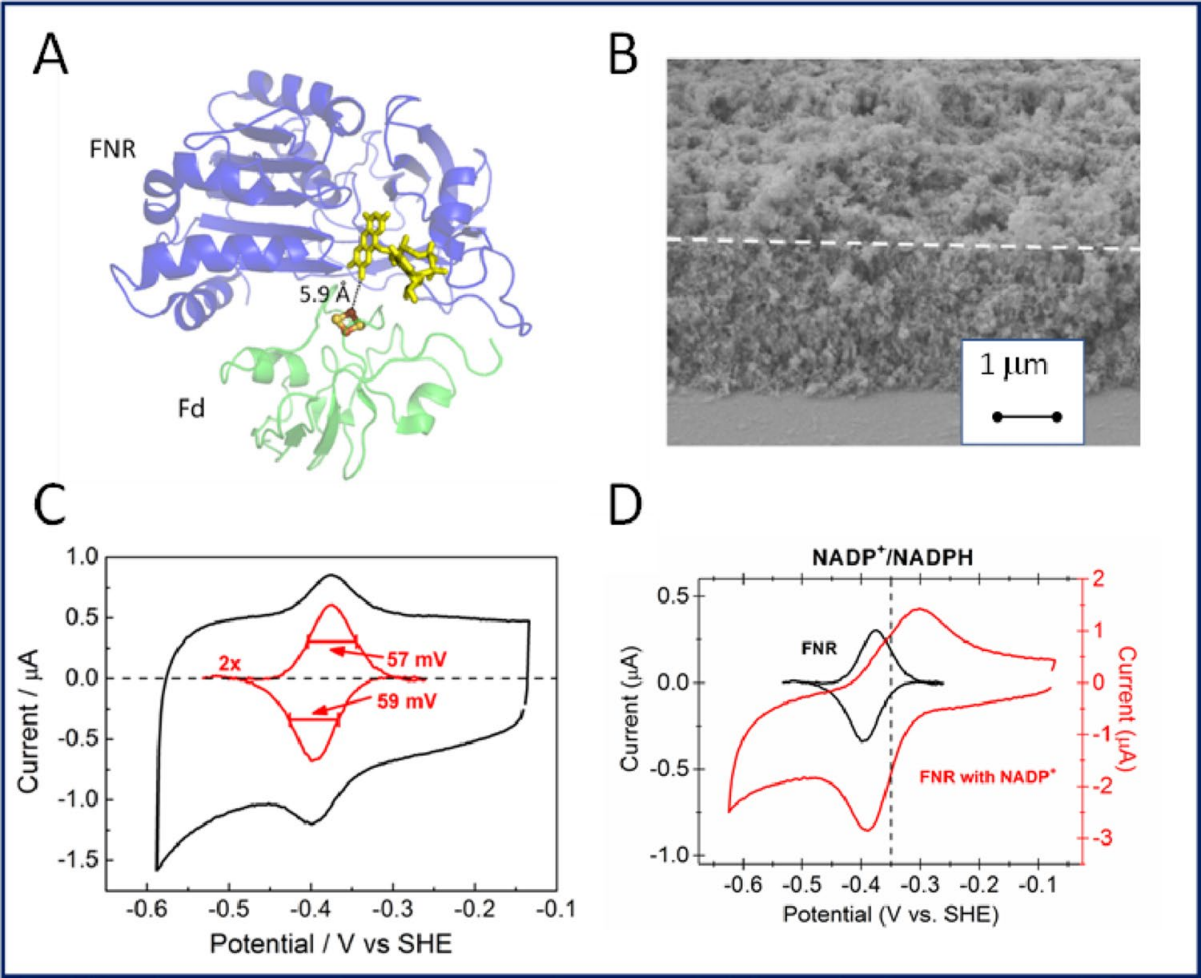
Key features of the e-Leaf platform. **A** Structure of the physiologically relevant complex formed between ferredoxin-NADP^+^ oxidoreductase (FNR) and a [2Fe-2S] ferredoxin (from 1GAQ) [243]. **B** Scanning electron microscopy (SEM) image of a layer of indium tin oxide (ITO) nanoparticles deposited on an ITO glass support [246]. **C** Non-turnover signal displayed by FNR at a porous ITO electrode [245]. **D** Catalytic voltammetry of FNR observed upon introducing NADP^+^ to the solution [106]

**Discovery and development.** From coupling *within* enzymes, we were to direct attention to coupling *between* enzymes [91]. Tandem catalysis facilitates multi-step reactions by linking sequential catalytic sites together, but to be effective each site should display high selectivity and activity. Nature does this naturally as enzymes are highly active and selective, and those catalysing a particular pathway are often organised in networks localised in confined microcompartments within living cells [240]. Although synthetic enzyme networks can be immobilised in templates or scaffolds, the overall process needs an energy input – light, chemical or electrochemical – and, ideally, must be capable of being controlled and monitored continuously [241, 242]. From 2017, my group started to develop a discovery we had made with the small (39 kDa) electron-transport enzyme ferredoxin-NADP^+^ oxidoreductase (FNR) [243]. This enzyme (Fig. 8A), found in chloroplasts and green algae, catalyzes the production of NADPH using photosynthetically-derived electrons mediated by ferredoxin [244].

Bhavin Siritanaratkul, then a graduate student in my lab, was exploring different opportunities afforded by porous indium tin oxide (ITO) electrodes (Fig. 8B). Tom Roberts, a 4th year undergraduate researcher, e-mailed me while I was visiting The University of Illinois to show me the results he had just obtained after introducing FNR to a mesoporous electrode prepared by coating ITO nanoparticles on graphite. A faint but clearly reversible non-turnover signal was present in the potential region expected for the active site FAD, so I immediately encouraged Tom to pursue this observation. He soon established the following: first, that the enzyme was a reversible electrocatalyst for NADP^+^/NADPH interconversion; second, that the narrow non-turnover signal was due to a cooperative two-electron process; third, FNR could be adsorbed at extremely high coverage (Fig. 8C) – implying that it becomes bound deeply within the electrode pores [245].

The scan-rate dependence of the peak-like catalytic cyclic voltammetry (Fig. 8D) suggested that NADP(H) was partially trapped in the ITO layer. Along with Bhavin Siritanaratkul and Clare Megarity, several students would soon join the e-Leaf team – graduates Giorgio Morello, Lei Wan, Beichen Cheng, and Ryan Herold, along with 4th year undergraduate researchers that included Ros Booth, Sarah Fitzpatrick and Adam Sills. Further experiments showed that the electrocatalytically generated NADP^+^ or NADPH (depending on the electrode potential applied) is internally recycled by a second enzyme – a dehydrogenase – once this also becomes adsorbed at the electrode [246]. The title of that paper ‘Electrocatalytic volleyball….’ implied the manner in which NADP(H) must be passed back and forth, often unproductively, between (and within) two groups (the ‘teams’) of enzymes, FNR and a dehydrogenase – ‘play’ being confined within a restricted space that made onward reaction at least as probable as escape. The way was thus open to drive enzyme cascades in a porous material, and highly engaging and productive collaborations were established with Nick Turner in Manchester and Chris Schofield in Oxford.

Porous electrodes have been studied for many years – through greatly increased surface area they allow higher catalyst loading, including incorporation of protein molecules [247–250]. Yet, strangely, the advantage afforded for tandem electrocatalysis is rarely mentioned [251, 252]. With the e-Leaf, it would now become possible to do far more – to study and exploit complex enzyme cascades with unprecedented ease and versatility. Electrophoretic deposition of commercial nanoparticles on conductive supports such as ITO glass, PGE and titanium foil quickly results in a mesoporous ITO layer between 3 and 10 microns deep [253]. All the evidence suggested that enzymes adsorb spontaneously and we reasoned that they must enter the pores to become active. The central requirement turned out to be the co-confinement of FNR (E1) and a dehydrogenase (E2) that uses the NADP(H) electrocatalytically generated by E1 to feed its own reaction, returning the cofactor back to E1. We soon established that the concentration of NADP(H) that is required (tens of micromolar in solution) is far lower than normally used in conventional biocatalysis procedures, and the reason for this would eventually become clear. Experiments carried out by Ryan Herold with E2 = isocitrate dehydrogenase 1 (IDH1 – an enzyme of the TCA cycle) proved very informative [254]. Human IDH1 purifies with 1–2 molecules of NADPH bound per dimer, which it carries into the electrode pores as cargo. The tiny quantity is sufficient to perform > 160,000 turnovers over a couple of days, without any additional cofactor being added. Using trumpet plot analysis Ryan could determine the amount of NADPH being recycled, and from the NMR quantification of enzyme-bound NADP(H), he could also estimate the amount of IDH1 present. It was thus concluded that FNR and IDH1 are concentrated in the pores to attain local levels approaching (or even exceeding) 1 mM: significantly, NADP(H) undergoes localised recycling and avoids escape.

**Fig. 9:**
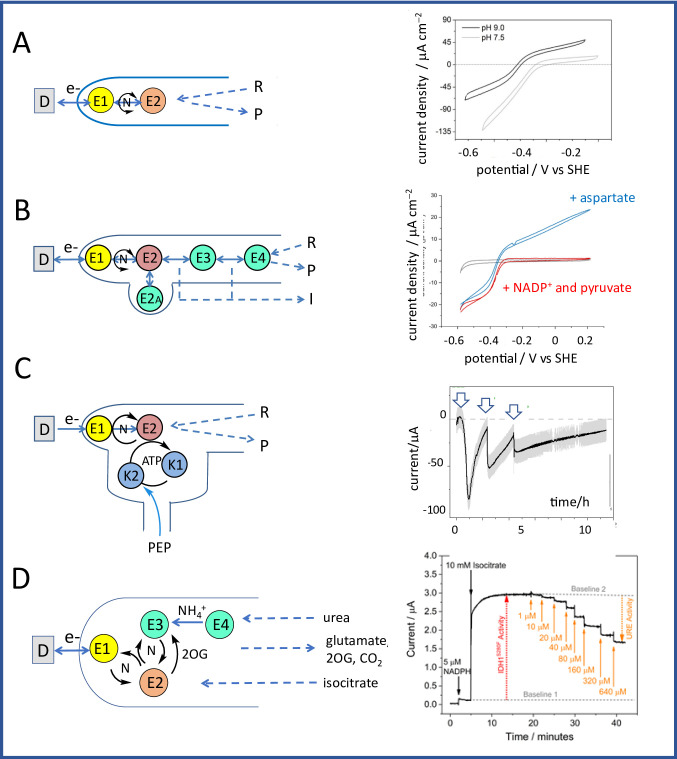
Examples of nanoconfined cascade extensions (see text for details). **A** The minimal pair: E1 is the transducing enzyme FNR which exchanges electrons with the metallic oxide by long-range electron tunnelling, and E2 is a NAD(P)(H)-dependent dehydrogenase [263]. **B** An extended linear cascade [256]. **C** A cascade that includes two kinases (K1 and K2) to regenerate ATP from AMP when phosphoenolpyruvate (PEP) is added (arrowed) [257]. **D** A cascade featuring an internal hydrogen-borrowing circuit, which responds immediately to injections of urea [267]

We introduced a version of cascade maps to emphasise nanoconfinement and input/output as shown in Fig. 9, in order to represent particular configurations. The dashboard, D, is the equipment through which inputs and outputs are controlled and processed: it includes potentiostat, computer, and equipment for delivering and removing reagents. Like the dashboard of a car it contains components that each have their analogous partner in electrochemistry – transmission (forward and reverse), accelerator, brake, odometer and navigation [255]. The enzymes are ordered in terms of their position in the reaction sequence, not their spatial positions (which are unknown). The ‘engine’ is the transducer, FNR (or an excellent substitute). The ability to anchor enzymes and (through channeling) control the escape of their products meant that once the parent E1-E2 pair is established, further enzymes, E3, E4… etc., representing all of the major classes, could be incorporated to build linear and branched cascades of increasing complexity [256, 257]. The concept and device were given the name the Electrochemical Leaf (e-Leaf) because the regeneration of NADPH by FNR resembles that occurring during the light-independent pathway of photosynthesis, although an advantageous difference is that the e-Leaf can drive oxidation as well as reduction simply by adjusting the electrode potential. The NAD(P)(H) cofactor and each of the intermediates along the sequence of enzyme-catalyzed reactions would now become current carriers, taking over from the electron which is only transferred at E1. The random distribution of pore sizes allows enzymes of various sizes to enter and be accommodated. In a typical case, we would apply to the electrode surface a mixture of enzymes in ratios designed to adjust for differences in their activity (inherent or experimentally apparent). Devices were made that were stable for several days and could be scaled up for synthesis using banks of electrodes constructed by depositing ITO or other metallic oxides on double-sided sheets of titanium foil [258, 259]. Following work by Medina and co-workers, we adopted a C-terminal variant of FNR that is active for NAD^+^/NADH interconversion, expanding the repertoire [260, 261]. In a detailed PFE investigation of the C354S variant, Megarity and co-workers showed how modifications to cofactor binding equilibria and kinetics are manifested in the FAD signals that are displayed [262].

It was soon established that the scope for the e-Leaf is very extensive, as bespoke cascades could be designed and manipulated with ease. The examples shown in Fig. 9 represent increasing complexity that is easily managed: they also include enzymes that depend on metal ions that are redox-inactive and may be labile and/or spectroscopically challenging. Figure 9A, featuring just the minimal E1-E2 pair, is the blueprint for myriad dehydrogenases – examples studied to date include where E2 = native and variant alcohol dehydrogenases (Zn, Mg) [259, 263], reductive aminase and imine reductase [246], and isocitrate dehydrogenase (Mg) [254, 264–266]. The reversible electrocatalytic CVs shown at the right are for an alcohol dehydrogenase at two different pH values.

The extended cascade represented in Fig. 9B consists of malate dehydrogenase (E2), fumarase (E3) and aspartate-ammonia lyase (E4), with carbonic anhydrase (Zn, E2A) generating CO_2_ in situ from bicarbonate arriving from solution [256]. In Fig. 9C, E2 is carboxylic acid reductase (CAR), which catalyses the reduction of carboxylic acids to aldehydes. This reaction is unfavourable with NADPH alone but is driven because CAR couples it to the hydrolysis of ATP to AMP: adenylate kinase (K1) and pyruvate kinase (K2) ensure in situ supply of ATP as the chemical fuel, phosphoenolpyruvate, is injected into the solution [257]. The cascade represented in Fig. 9D features an internal hydrogen-borrowing circuit in which glutamate dehydrogenase (E3) competes with FNR (E1) for the NADPH generated by isocitrate dehydrogenase (Mg, E2), provided NH_4_^+^ is present along with the other substrates [267]. Notably, the current responds immediately to injections of micromolar concentrations of urea which is hydrolysed by urease (Ni, E4) to form NH_4_^+^ and CO_2_: thus, under nanoconfinement, the collective action of four enzymes appears virtually synchronous.

`The fast and highly channelled flow of reactions depends on the nanoconfinement and the high activities and selectivities of the different enzymes, which makes escape less likely than onward processing. Enzymes do not need to be in closest proximity, and within the microns-deep layer the depth to which an enzyme is located is not restrictive [268, 269]. Considering the current to be the information carrier, the upstream direction (whereby the cascade reaction flows ‘upstream’ from the final enzyme to FNR) differs from the downstream direction (whereby the cascade reaction begins at FNR) – the former but not the latter being directly dependent on the activity of enzymes along the chain [256]. End products escape most readily as there is no enzyme to process them further: these are detected and characterised by analytical methods, particularly NMR. The transduction between electrical current and channeled chemical reaction flow via NAD(P)(H) means that apart from E1 and E2, further enzymes of the cascade do not need to catalyse a redox reaction in order for them to become electroactive [266]. The action of an inhibitor of an upstream enzyme is not only easily observable, but the reaction can be directly tracked in time or reversed by removing the inhibitor – advantages we exploited to investigate the mechanism of a drug that acts as a slow, allosteric inhibitor of a cancer-linked variant of isocitrate dehydrogenase [264, 265].

**Scope and future of the e-Leaf.** The e-Leaf brings together diverse scientific directions, including materials, nanotechnology, tandem/cascade biocatalysis, electrochemistry, enzyme kinetics, biophysics, nanoconfinement, the collective properties of crowded enzymes, the indirect rendering of electroactivity on non-redox enzymes and visionary potential for commercial applications [270]. The special environment offers interesting opportunities and challenges for spectroscopic methods such as EPR [271]. The hydrophilic mesoporous layer produced by electrophoretic deposition of metallic oxide nanoparticles has clear advantages over well-known framework materials (zeolites, MOFs and COFs) through the natural creation of large, irregular spaces, suited for hosting enzyme molecules of varying size and shape, combined with inherent electronic conductivity [272].

As a further example of bio-inspired catalysis – a term normally applied to copying Biology’s tricks at a molecular level – the e-Leaf directs our attention to the efficient containment of sequential catalytic reactions through nanoconfinement, and if and how we could mimic the way that the enzymes of cascades are concentrated, crowded and organised in living cells [273–276]. The e-Leaf supports the view that biological NAD(P)(H) recycling can be highly localised, instead of usually being assumed to exist as a delocalised pool [277]. Would it be easy to replace enzymes with small-molecule electrocatalysts? Enzymes have two overwhelming advantages: (1) their high activities greatly increase the probability that a reacting species will be processed before it can escape, and their high selectivities ensure fidelity at each step; (2) their large size allows them to become physically trapped and crowded in spaces that are still large enough to allow relatively free movement of reacting species. Small molecular catalysts must be covalently immobilised [278]. The speed (seconds) with which information is transmitted through complex sequences of reactions (as in living cells) led us to introduce the term ‘cascadetronics’, thereby emphasising the immediate and interactive control of entire enzyme cascades that now becomes possible [267]. The analogy with electronics helps to reinforce the emerging ideas about enzymes acting as logic gates, put forward by Katz and others [279, 280].

Commercialisation has never been my ultimate goal, but the e-Leaf has potential applications ranging from efficient production of valuable fine chemicals and pharmaceuticals to enzyme and drug development, including detailed mechanistic investigations [258, 259, 264, 265, 281]. The electrodes can be scaled up for synthesis or down for multiplexing. Confinement of enzyme cascades inside electrically conducting materials could lead to novel applications where conventional external electrolyte is unnecessary. How these opportunities are taken up depend not only on identifying niche applications, but also on the willingness of companies and investors to think ‘electrochemically’ [282, 283].

## Final notes

This extended essay has summarised over 35 years of research in which I and others have looked at redox proteins and enzymes from a complementary and very different angle to the spectroscopists and crystallographers with whom we have worked closely. To make sense of complex biological redox reactions, it is necessary to monitor enzymes in action while asking questions in dialogue fashion, and PFE offers this special interaction – revealing how electron transfer is coupled in time, space and thermodynamics to exquisite chemical processes. Drawing on many disciplines increases the probability of making a serendipitous discovery – an example being the reversible electrocatalysis of localised NADP(H) recycling by FNR trapped in a porous material. The pictures provided by PFE may be likened to collages – views of numerous different images (individual pieces of evidence) pasted together, thereby revealing direct coupling and more remote relationships that are difficult to visualise individually.

I became President of SBIC in 2004, taking over from Harry Gray. I passed the baton on to Bob Scott in 2006. I finish with my last President’s message, written in May 2006 and appearing in JBIC, when I sadly announced the passing of a visionary scientist, Antonio Xavier, a founding father of the ‘BICs’.

*This is my last contribution to ‘President’s Note’ as I pass on the reins to Bob Scott. Suffice to say that I have very much enjoyed chairing the regular*,* cordial meetings and correspondence with all the Council members*,* and look forward to serving two more years on Council as President-Past. My predecessor Harry Gray can now relax !*

*Some very sad news – Antonio Xavier passed away on May 7 after a long illness borne with the characteristic courage and spirit that so marked him throughout his life. Antonio will be greatly missed by all of us who have had the privilege of knowing him and interacting with him*,* whether it be discussing an idea or receiving so freely his wisdom and generosity. Earlier this year it was announced that he would be the recipient of the 2006 Eurobic Medal and of course*,* under these circumstances*,* a posthumous presentation will be made in Aveiro.*

*Finally*,* and to leave on a happier note*,* we are anxious to start receiving nominations for the ‘SBIC Young Scientist Award’ the first of which will be presented next year at ICBIC 13 in Vienna. Details are to be found on the SBIC website. You have until December 31. Please don’t forget !*

*Best wishes to you all.*

*Fraser*.

## Data Availability

All data used in this paper have been published and cited.
